# Structural Elucidation and Activities of *Cordyceps militaris*-Derived Polysaccharides: A Review

**DOI:** 10.3389/fnut.2022.898674

**Published:** 2022-05-31

**Authors:** Miao Miao, Wen-Qian Yu, Yuan Li, Yan-Long Sun, Shou-Dong Guo

**Affiliations:** Institute of Lipid Metabolism and Atherosclerosis, Innovative Drug Research Centre, School of Pharmacy, Weifang Medical University, Weifang, China

**Keywords:** *Cordyceps militaris*, polysaccharide, structure-activity relationship, bioactivity, mechanisms of action

## Abstract

*Cordyceps militaris* is a parasitic edible fungus and has been used as tonics for centuries. Polysaccharides are a major water-soluble component of *C. militaris*. Recently, *C. militaris*-derived polysaccharides have been given much attention due to their various actions including antioxidant, anti-inflammatory, anti-tumor, anti-hyperlipidemic, anti-diabetic, anti-atherosclerotic, and immunomodulatory effects. These bioactivities are determined by the various structural characteristics of polysaccharides including monosaccharide composition, molecular weight, and glycosidic linkage. The widespread use of advanced analytical analysis tools has greatly improved the elucidation of the structural characteristics of *C. militaris*-derived polysaccharides. However, the methods for polysaccharide structural characterization and the latest findings related to *C. militaris*-derived polysaccharides, especially the potential structure-activity relationship, have not been well-summarized in recent reviews of the literature. This review will discuss the methods used in the elucidation of the structure of polysaccharides and structural characteristics as well as the signaling pathways modulated by *C. militaris*-derived polysaccharides. This article provides information useful for the development of *C. militaris*-derived polysaccharides as well as for investigating other medicinal polysaccharides.

## Introduction

Cordyceps species have been used as medicine, tonics, and food for centuries in many countries (1, 2). Approximately 750 Cordyceps species are mainly distributed in Asia, Europe, and North America (1). *Cordyceps militaris* (Yong Chong Cao, 蛹虫草) is a well-developed Cordyceps species (3, 4). Pharmacological studies have suggested that artificially cultivated *C. militaris* is useful against many diseases, especially non-communicable diseases (5–7). Some medicinal and tonic products of *C. militaris* have been developed and commercialized around the world, especially in Asian countries (3, 8). The annual output value of *C. militaris*-derived products is estimated to be 10 billion RMB in China (9).

The primary metabolite, polysaccharide, is one of the major water-soluble bioactive components of *C. militaris* (3, 6). Compared to the secondary metabolites, *C. militaris*-derived polysaccharides have not been well characterized (1, 2, 4, 10–12). Four years’ ago, Zhang et al. reviewed the extraction, isolation, purification, structural characteristics, and bioactivities of *C. militaris*-derived polysaccharides (3). The pharmaceutical mechanisms of *C. militaris* polysaccharides including antioxidant, immunomodulatory, and anti-tumor activities have also been reviewed recently (7). The activity of the polysaccharide is determined by its monosaccharide composition, molecular weight (Mw), glycosidic linkage, and degree of branching. With increasing use of advanced analytical tools, the structural characterization of polysaccharides has improved greatly during the past several years. However, the structural characteristics of these polysaccharides in relation to some bioactivities such as anti-diabetic, and anti-hyperlipidemic, and anti-atherosclerotic effects have not been well summarized. Furthermore, these is a lack of graphic representations that clearly show the reaction processes involved in structural elucidation and the signaling pathways mediated by *C. militaris*-derived polysaccharides. This is the motivation to review the advances in the methods used for structural elucidation and to further describe the structural characteristics and the signaling pathways that are modulated by *C. militaris*-derived polysaccharides. In this article, we review the related literature mainly from the year of 2019 to the present that were obtained as search results from PubMed using “*C. militaris* and polysaccharide” or “mass spectrometry and polysaccharide” as keywords.

## Methods for Elucidation of Polysaccharide Structure

### Fourier Transform Infrared Spectrometry

Except for chemical methods, Fourier transform infrared (FT-IR) spectrometer is a common and easily available tool to quickly identify polysaccharides. In FT-IR spectrum, a strong U-type band observed at approximately 3,400 cm^–1^ represents the typical O-H stretching vibration, and the weak bands at around 2,930 cm^–1^ represent the typical C-H stretching vibration. Furthermore, the strong band presents at approximately 1,050 cm^–1^ is arisen from the C-O-C glycosidic bond vibration, while the band at around 1,600 and 1,655 cm^–1^ can be assigned to C = O stretching vibration and the bending vibration of -NH- (-CONH-), respectively (6). The peak at approximately 1,250 cm^–1^ suggests the presence of sulfate in axial position. Of importance, the bands between 1,000 and 800 cm^–1^ are useful for determination of α- and β-configurations of glycosyls. For instance, the bands at around 880 cm^–1^ suggest the potential presence of mannosyl and galactosyl residues in a β-configuration; and the bands at approximately 810 and 850 cm^–1^ may arise from α-D mannosyl and α-D glucosyl residues, respectively (13–16).

### Monosaccharide Composition Determination

**FIGURE 1:**
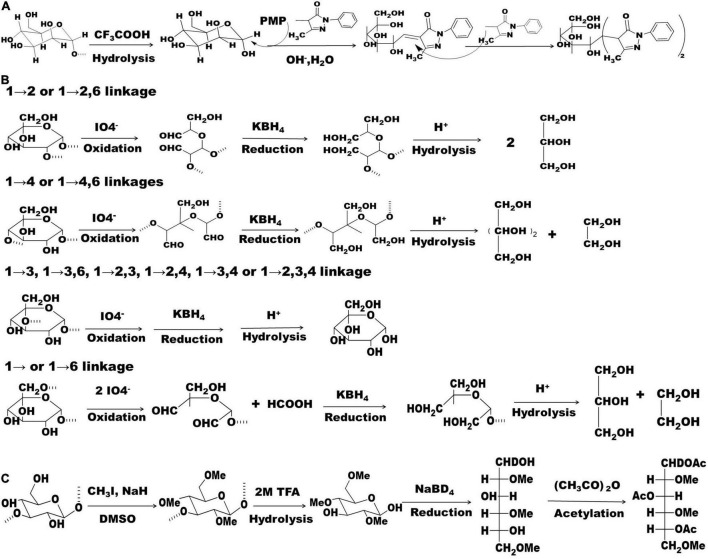
Chemical reactions related to PMP derivatization, periodate oxidation-smith degradation, and methylation. **(A)** Polysaccharide hydrolysis and monosaccharide derivatization with PMP; **(B)** chemical reactions of periodate oxidation-Smith degradation; **(C)** chemical reactions that involved in polysaccharide methylation. PMP: 1-phenyl-3-methyl-5-pyrazolone.

To accurately determine monosaccharide composition, a complete acid hydrolysis of polysaccharide is needed. Theoretically, all strong acids can be used to release monosaccharides at a concentration of 2–4 mol/L. Considering the next neutralization step, trifluoroacetic acid (TFA) with a good volatility is the most popular acid because it can be easily removed by a rotary evaporator (17, 18). In brief, approximately 10 mg of polysaccharide dissolved in 1–2 mL of 2.0 mol/L of TFA is put into a 5 mL ampule container, which is sealed and maintained at 110°C for 6 h. Next, neutral monosaccharides can be detected by gas chromatography (GC) after the monosaccharides are converted into acetylated aldononitrile derivatives, however, acidic monosaccharides need to be converted into their corresponding alditols by an appropriate reductant, such as sodium borohydride, before chemical derivatization (14). Compared to GC, high-performance liquid chromatography (HPLC) is more popular because this method can detect all the reducing monosaccharides (neutral, acidic, and basic) after monosaccharide derivatization with 1-phenyl-3-methyl-5-pyrazolone (PMP) (Figure 1A). Moreover, HPLC combined with PMP derivatization can detect more than ten kinds of monosaccharides with a high sensitivity and resolution in a single run (16, 19). Ion chromatography is another method for determining monosaccharide composition without any kind of derivatization. However, some natural monosaccharides, such as galactose/N-acetyl glucosamine or mannose/xylose, have comparably bad resolution on ion chromatography (16). Recently, an HPLC-tandem mass spectrometry (HPLC-MS/MS) method was developed for simultaneous detection of 17 monosaccharides including aldoses, ketoses, amino sugars, and uronic acids, in the multiple reaction monitoring mode after aldononitrile acetate derivatization (20). Additionally, GC-mass spectrometry (GC-MS) can be used for determination of the absolute configuration (D or L) of monosaccharides (21).

### Glycosyl Linkage Determination

Periodate oxidation-Smith degradation is a traditional method used to assess glycosidic linkages (8, 22). Glycosyls with distinct linkages may produce different reaction products (Figure 1B), which can be analyzed by thin layer chromatography and GC. However, it is impossible to assign glycosidic linkage of a polysaccharide that is composed of more than one type of monosaccharide (13). Therefore, this method has been gradually replaced by methylation and nuclear magnetic resonance (NMR) analysis.

### Methylation Analysis

Methylation analysis is a classical and putative method used to determine glycosidic linkages (Figure 1C). In brief, approximately 2–3 mg of polysaccharide is needed to perform this assay. During the reaction process, anhydrous conditions and protection with nitrogen are strongly suggested. FT-IR is generally used to evaluate the completeness of methylation. In an FT-IR spectrum, the absence of the U-type peak at approximately 3,400 cm^–1^ and the significant increase of C-H stretching variation at around 2,930 cm^–1^ indicate a complete methylation reaction (15, 16). Next, permethylated polysaccharide is completely hydrolyzed as mentioned in the monosaccharide composition analysis above. The resulting hydrolysates are reduced with sodium borodeuteride (NaBD_4_), and then acetylated with acetic anhydride. To keep in alignment with the database, use of sodium borohydride (NaBH_4_) as a reductant is not encouraged. The final methylated alditol acetates are generally analyzed using GC-MS (16). The open accessed Complex Carbohydrate Structure Database created by the Complex Carbohydrate Research Center of the University of Geogia^1^ is generally used to explain the GC-MS data, thereby determining glycosidic linkages. A comparison of the total amount of branched glycosyls with the total amount of glycosyls that derived from the non-reducing end is useful in evaluating the correctness of the methylation analysis. However, polysaccharides with substitutions, such as *O*-methyl, are not considered in this type of comparison (16). Recently, an ultra-HPLC-MS/MS method was developed for the rapid determination of glycoside linkages of polysaccharide and oligosaccharide in a multiple reaction monitoring mode. The permethylated samples are hydrolyzed and derivatized with PMP before analysis and the resulting linkage profiles can be determined and quantified by the library containing 22 kinds of glycosidic linkages that are built using oligosaccharide standards (23).

### Nuclear Magnetic Resonance Analysis

NMR spectroscopy is another powerful tool for determination of glycosidic linkages of carbohydrate. One-dimensional (1D-) (^1^ H-, and ^13^C-NMR) and two-dimensional (2D-) NMR experiments, including distortionless enhancement by polarization transfer spectroscopy (DEPT), ^1^H-^1^H correlated spectroscopy (COSY), ^1^H-^13^C heteronuclear multiple quantum coherence spectroscopy (HMQC), ^1^H-^13^C heteronuclear multiple bond correlation spectroscopy (HMBC), total correlation spectroscopy (TOCSY), and nuclear overhauser effect spectroscopy (NOESY), are usually performed for accurately determining glycosidic linkages. In the 1D-NMR spectra, the anomeric signals always display in down-field regions, which could provide useful information for determining distinct glycosyls and even for quantitative analysis of the various glycosyls. However, the rest signals (H2-H6 or C2-C5) of heteropolysaccharides usually overlap with each other in the ^1^H- and ^13^C-NMR spectra, which makes it hard for a correct assignment. 2D-NMR experiments play key roles in structural elucidation of polysaccharides. ^1^H-^1^H COSY or TOCSY can give valuable information on the associated protons within a sugar ring. In general, the correlation between H1-H3 is easily identified even in the ^1^H-^1^H COSY spectrum of a heteropolysaccharide. However, it is difficult to accurately assign the correlation between H3-H6 due to the large degree of overlap of these proton signals in the ^1^H-NMR spectrum. An HMQC experiment is valuable in determining the direct correlation between protons and carbons, such as H_1_/C_1_ or H_3_/C_3_. In comparison with ^13^C-NMR spectrum, DEPT spectrum can provide useful information of *O*-6 substituted signals, which show inverted peaks at approximately 66 ppm. The long-range couplings (within 3 bonds) between carbon and proton signals in the HMBC spectrum are useful in making up the missing correlations within a sugar ring and even those between the connective glycosyls. Furthermore, NOESY and TOCSY provide complementary information based on bond connectivity. The solvent and relaxation rates of the protons can significantly influence the sensitivity of these correlation signals (24). Collectively, HMBC, NOESY, and TOCSY spectra are important in the construction of glycosyl connections.

Given the complex linkages of glycosyls and heavy overlap of NMR signals, it is virtually impossible to assign all the NMR signals for most of the polysaccharides. However, some NMR signals can be assigned in combination with methylation analysis and using the available literature. Furthermore, NMR data are useful for determining the α- and β-configurations of glycosyls based on carbon-proton spin-coupling constants (25). As for the D-type pyranosyls in the ^4^C_1_ conformation, a ^1^J_*C*1,H1_ at ∼170 *Hz* indicates an α-anomeric sugar configuration, and a ^1^J_*C*1,H1_ at ∼160 *Hz* suggests a β-anomeric sugar configuration (16). Alternatively, anomeric proton signals in the region of 5.60–4.90 and 4.90–4.30 ppm may be assigned as α- and β-anomers, respectively. It seems that anomeric protons of galactosyl in a β-D configuration does not match the latter rules. However, anomeric carbons in galactosyls in the β-D configuration always show a downfield chemical shift in ^13^C-NMR spectrum (greater than 104 ppm in general), which is useful to determine the α- and β-configuration of these galactosyls (15, 16). Of importance, an accurate monosaccharide composition analysis is the basis for assignment of glycosyl linkage patterns.

The NMR signals of homopolysaccharides with simple glycosyl linkages are easily assigned. For instance, the structural characteristics of the *C. militaris*-derived β-D-(1→6)-glucan and the glucan mainly consisted of →4)-α-D-Glc*p* (1→ glycosyls are elucidated by our lab (14, 15). Furthermore, glycosyls in a specific configuration and linkage pattern usually show similar chemical shifts in NMR spectra. For example, the galactosyls in the β-D-configuration may present as the side chains in *C. militaris*-derived polysaccharides in the form of →2)-β-D-Gal*f* (1→ and/or β-D-Gal*f* (1→ glycosyls, whose anomeric carbon generally present at approximately 106 ppm in the ^13^C-NMR spectrum (15). The anomeric signals of →2,6)-α-D-Man*p* (1→, α-D-Man*p* (1→, and →6)-α-D-Man*p* (1→ glycosyls are generally present at approximately 100.5, 102.0, and 98.0 ppm, respectively. Furthermore, O-methyl is also found in *C. militaris*-derived polysaccharides, and this kind of methyl shows a chemical shift at approximately 54.0 ppm in ^13^C-NMR spectra (15, 26). The new magnets beyond 1 G*Hz* have greatly enhanced the sensitivity and resolution of the NMR signals, and the ^13^ C-, ^15^ N-, and ^19^F-labeling strategies can further improve the sensitivity and resolution (27). Solid-state NMR spectroscopy in combination with magic angle spinning techniques can provide detailed signals in a non-destructive manner. This method has also been used to determine the structure of polysaccharides in different forms and even in plant and cell walls (28–31). The application of solid-state NMR has been reviewed recently by different teams (32–34).

### Mass Spectrometry

**FIGURE 2:**
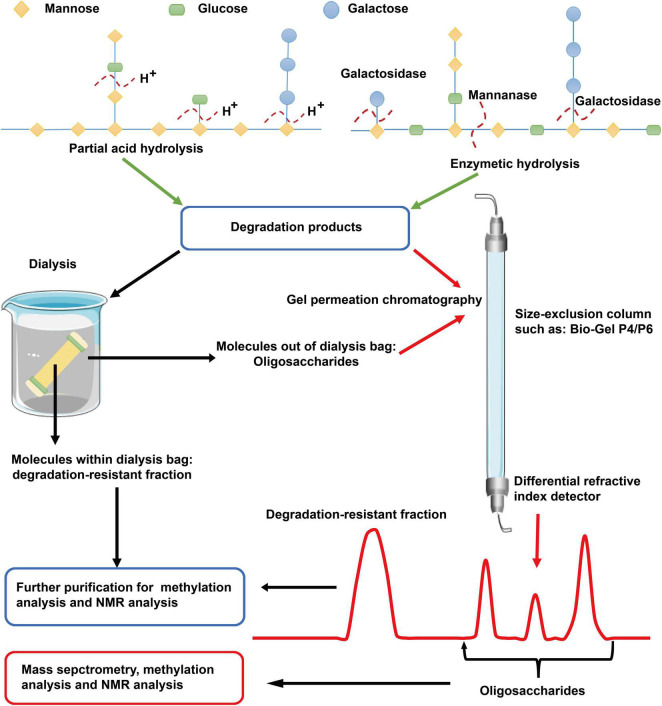
Scheme for elucidation of polysaccharide structure based on degradation. Partial acid hydrolysis with low concentrations of TFA is generally used to remove degradable side chains of polysaccharides. Enzymatic hydrolysis with glycosidase is a more specific method for degradation of glycosyls. The released oligosaccharides and the degradation-resistant fraction can be separated by dialysis using dialysis bags with Mw cutoff < 5.0 kDa or size-exclusion columns such as Bio-Gel P4 or P6. The obtained products can be further analyzed by mass spectrometry, methylation analysis, and NMR experiments. TFA, trifluoroacetic acid; NMR, nuclear magnetic resonance.

Hydrolysis is helpful in elucidating the structure of polysaccharide (Figure 2). Partial hydrolysis with TFA at a concentration of < 0.1 M (such as 0.05 M) is generally used to remove degradable side chains of polysaccharides (35). The released oligosaccharides and the resistant backbone can be further analyzed by methylation and NMR experiments (26). Data obtained before and after partial hydrolysis are usually combined to elucidate the fine structure of a polysaccharide. Enzymatic hydrolysis is another valuable method for determining glycosidic linkages in polysaccharides. Based on enzymatic digestion (such as, α-amylase, β-glucanase, arabinanase, xylanase, galactanase, and pectinase), →4)-α-D-Glc*p* (1→, →4)-β-D-GlcA*p* (1→, and →4)-α-D-Gal*p* (1→ glycosyls are found to exist in wild and cultured *C. militaris* and other species of Cordyceps (17, 18, 36). The saccharide mapping profiles obtained in combination with high performance thin layer chromatography is valuable in distinguishing polysaccharides from different species of Cordyceps (17, 36). Of importance, the hydrolyzed products such as oligosaccharides and low Mw polysaccharides can be further analyzed by mass spectrometry (MS). As reviewed previously, matrix-assisted laser desorption/ionization (MALDI)-MS and HPLC-MS/MS with a high sensitivity are utilized for the structural elucidation and quantification of oligosaccharides and polysaccharides with a low Mw (17, 18, 37).

The saccharide analysis can be carried out using electrospray ionization MS (ESI-MS) and MALDI-time of flight (MALDI-TOF) MS. Collision-induced dissociation is widely applied for detection of oligosaccharide (38). The ion fragments are informative for explaining the glycosyl linkages of carbohydrates, and the systematic nomenclature rules have been previously documented by Domon and Costello (39). Of note, monosaccharide loss, migration, and rearrangement, during MS analysis is common due to the weak glycosidic bonds. Permethylation is an efficient method to improve the stability of glycans. Hydrophilic interaction chromatography coupled with mass spectrometric detection (HILIC-MS) is also used to sequence carbohydrates (40, 41). Moreover, HPLC-MS was recently used to detect the metabolites of polysaccharides as well as their interaction with proteins (42). Ionization efficiency, instability, and limited sensitivity of molecules with low Mw are the major factors that influence the application of MALDI-TOF mass spectrometry for the analysis of carbohydrates. The compound, 2-hydrazinoquinoline, can react with the reducing end of carbohydrates with high stability and efficiency. This derivatization method can effectively improve the problems of MALDI-TOF mass spectrometry as mentioned above (43). Mass spectrometry imaging is a novel technique used for investigating the distribution of metabolites in plant tissues. For instance, MALDI-TOF MS imaging has been used to reveal the disaccharide distribution in onion bulb tissues and the chemical components of *C. sinensis* (44, 45). Of note, microarray in combination with MALDI-TOF mass spectrometry was recently developed for the detection of glycosphingolipid glycans (46).

## Structural Characteristics of *Cordyceps militaris*-Derived Polysaccharides

**FIGURE 3:**
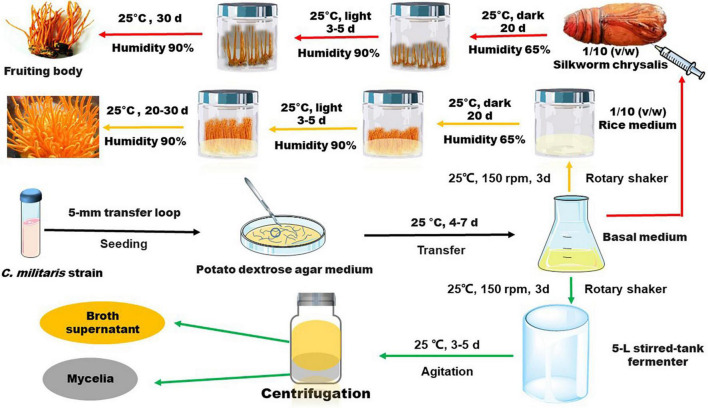
General methods used for fermentation and cultivation of *C. militaris*. The strain is first cultivated in dishes or test tubes with potato dextrose agar medium, and then it is transferred to liquid medium for a further cultivation. Several days later, the obtained liquid medium is used for an amplified fermentation to obtain mycelia and supernatant through centrifugation. Alternatively, the strain in liquid medium is seeded into solid media (such as rice medium) or be injected into silkworm chrysalis for further incubation to obtain *C. militaris* fruiting bodies.

**FIGURE 4:**
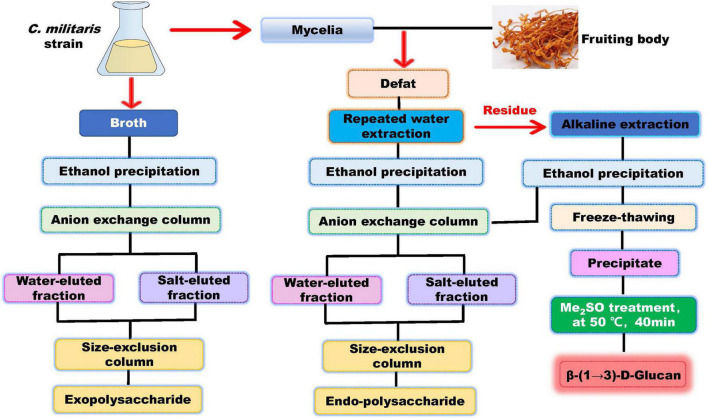
Scheme for Extraction and Purification of *C. Militaris*-Derived polysaccharides.

The methods used for cultivation of *C. militaris* are shown in Figure 3. *C. militaris*-derived polysaccharides can be obtained from mycelia fermentation (mycelia and fermentation broth) and cultivated fruiting bodies. Fermentation broth can be used to obtain extracellular polysaccharide (exopolysaccharide), while mycelia and fruiting bodies of *C. militaris* are used to extract intracellular polysaccharide (endo-polysaccharide). The isolation and purification processes for preparation of *C. militaris*-derived polysaccharides are summarized in Figure 4. Generally, only polysaccharides with high purity are used for the analysis of structural characteristics. Most of the polysaccharides derived from *C. militaris* are consisted of glucose (Glc), galactose (Gal), and mannose (Man) (3). However, polysaccharides containing other monosaccharides, such as rhamnose, arabinose (Ara), xylose (Xyl), ribose, fucose, galacturonic acid (GalA), glucuronic acid (GlcA), and N-acetyl galactosamine, can also be isolated from *C. militaris* (3, 18).

### Mycelia Fermentation-Derived Polysaccharides

The general fermentation processes are summarized in Figure 3. The fermentation conditions and the proposed mechanisms for biosynthesis of exopolysaccharide in Cordyceps have been reviewed recently by Yang et al. (47). Repeated batch cultivation is reported to enhance exopolysaccharide production of *C. militaris* (48). UV light irradiation-induced mutagenesis is found to improve production of extracellular and intracellular polysaccharides by more than 120-fold (49, 50). Ionic conditions and pH of the culture media also have important effects on the structural characteristics of polysaccharides. Addition of metal ions is found to influence the production of exopolysaccharides, such as Mg^(2+)^ and Mn^(2+)^ can improve while Ca^(2+)^ and K^(+)^ may reduce the yield (51, 52). Addition of Na_2_SO_4_ may induce the presence of sulfate in polysaccharides obtained *via* fermentation (53). Furthermore, addition of metal ions in the culture medium may result in metal ion-enriched polysaccharides. For instance, addition of FeSO_4_ solution can promote formation of polysaccharide-iron (III) complexes containing 2.73% of iron (54). The pH of the culture medium is reported to influence gene expression of mycelia, thereby modulating structural characteristics of polysaccharides extracted from fermented mycelia (55). Of note, a weak alkaline (pH 8–9) culture medium is reported to increase production of β-(1→6)-glucan (55). Recently, gene engineering strategies have also been applied to modulate production of *C. militaris* polysaccharides. For instance, the combined overexpression of phosphoglucomutase and UDP-glucose 6-dehydrogenase genes can increase production of exopolysaccharides by 78.13% compared to that of wild-type strain (56). Furthermore, bacteria in sclerotia have been demonstrated to influence mycelium biomass and metabolites of *C. militaris* (57). A recent study showed that submerged fermentation with talc microparticles can promote polysaccharide production (58).

**FIGURE 5:**
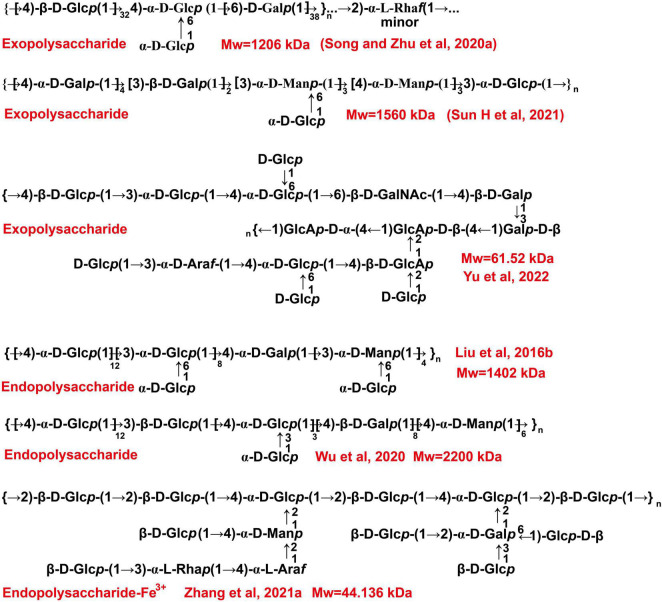
The presumed chemical structures of polysaccharides derived from the fermentation broth of *C. militaris*.

Based on the literature, we presumed some chemical structures of fermentation-derived *C. militaris* polysaccharides as shown in Figure 5. Two purified exopolysaccharides have been extracted from *C. militaris* strain (CICC 14015), and they have a similar Mw of more than 1,000 kDa. However, their monosaccharide composition and glycosidic linkages are completely different (59, 60). The exopolysaccharide obtained from *C. militaris* strain (CICC 14014) is different from that of CICC 14015. As reported, this heteropolysaccharide with a lower Mw has →4)-α-D-Glc*p* (1→ and →4,6)-α-D-Glc*p* (1→ glycosyls as its main chain (61). Another heteropolysaccharide mainly consisting of →2)-α-D-Man*p* (1→ and →6)-α-D-Man*p* (1→ glycosyls is obtained from *C. militaris* strain (KCTC 6064) (62). Most of the endo-polysaccharides obtained from the fermented mycelia are composed of at least three kinds of monosaccharides. Of note, most of them are mainly composed of glucose and have a backbone consisted of →4)-α-D-Glc*p* (1→ glycosyls (22, 63–65). The backbone of some heteropolysaccharides is found to be consisted of →2)-α-D-Man*p* (1→ glycosyls (8).

### Structural Characteristics of Polysaccharides Obtained From the Fruiting Body of *Cordyceps militaris*

**FIGURE 6:**
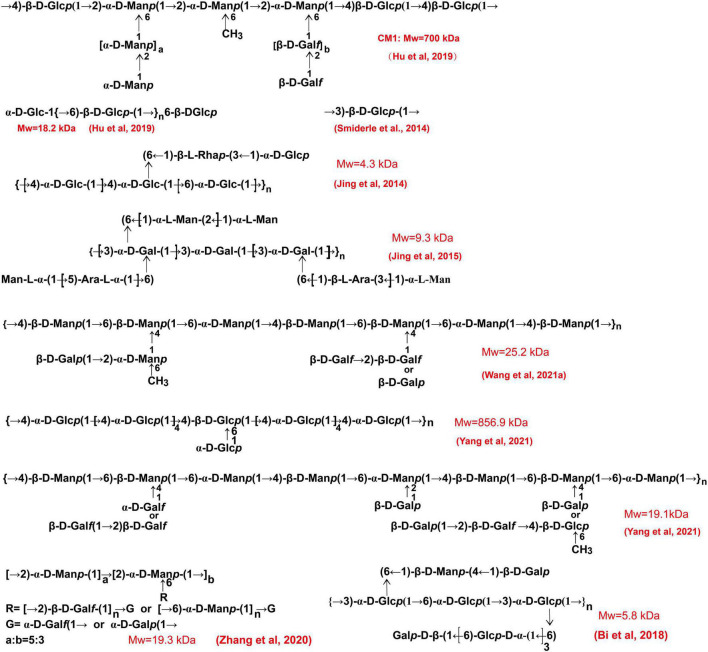
The chemical structures of polysaccharides derived from the fruiting body of *C. militaris*. These structures are obtained from related literature.

**FIGURE 7:**
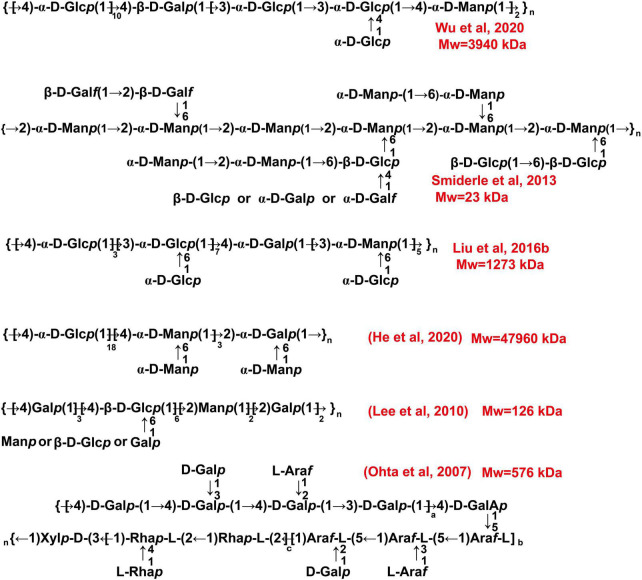
The chemical structures of polysaccharides derived from the fruiting body of *C. militaris*. We presumed these structures according to the descriptions in the literature.

The general processes for cultivation of the fruiting body of *C. militaris* are shown in Figure 3. Many factors, such as light irradiation and intensity, can influence polysaccharide production of the fruiting bodies (50, 66). Spaying biotic elicitors, such as chitosan (1 mg/L), can increase yield of polysaccharide by 1.41-fold (67). Furthermore, culture time also affect the production of polysaccharides. Within 35–45 days, polysaccharide content increases gradually and declines with a prolonged culture time (68). Recently, a comprehensive transcriptomic analysis of *C. militaris* cultivated on germinated soybeans was carried out by Yoo et al. (69). The gene information obtained from the analysis is useful in modulating the production of metabolites. In this article, we summarize the presumed structures of the *C. militaris* fruiting body-derived polysaccharides that are provided by the related literature (Figure 6). We also presume some chemical structures of polysaccharides according to the descriptions in the literature as shown in Figure 7.

As Glc is the major component of the cell walls of *C. militaris*, polysaccharides mainly composed of Glc are the most documented carbohydrate complex. A recent study demonstrated that →4)-α-D-Glc*p* (1→ linked glucan is specific to Cordyceps caterpillar and can be used as a marker for Cordyceps (70). Indeed, many water-extracted polysaccharides of the fruiting body of *C. militaris* have →4)-α-D-Glc*p* (1→ glycosyls as their backbone (22, 62, 64, 71, 72). Furthermore, most of these polysaccharides are heteropolysaccharides. However, several groups isolated glucans from the fruiting body of *C. militaris* (14, 73). Our group obtained a linear →6)-β-D-Glc*p* (1→ glucan with a Mw of 18.2 kDa (14). Some heteropolysaccharides are found to contain →4)-β-D-Glc*p* (1→ and →6)-α-D-Glc*p* (1→ glycosyls (14, 72). Polysaccharides mainly consisting of →2)-α-D-Man*p* (1→ glycosyls are also found in the fruiting bodies of *C. militaris* (3, 19).

The alkaline extracted polysaccharides are also reported. For instance, an alkali-extracted polysaccharide is found to have →2)-α-D-Man*p* (1→ and →2,6)-α-D-Man*p* (1→ glycosyls as its backbone (74). The polysaccharide CMPB90-1 obtained *via* 0.3 mol/L of NaOH is mainly composed of →6)-α-D-Glc*p* (1→ and →3)-α-D-Glc*p* (1→ glycosyls (75). Our lab obtained a novel alkali-extracted polysaccharide which mainly composed of →4)-β-D-Man*p* (1→, →6)-β-D-Man*p* (1→, and →6)-α-D-Man*p* (1→ glycosyls (16). Of note, alkaline-extracted (5% KOH) polysaccharides can be further treated with a free-thawing process, and the water insoluble fractions can be further treated with Me_2_SO to obtain a β-(1→3)-D-glucan (76). Our team obtained a glucan with →4)-α-D-Glc*p* (1→ and →4,6)-β-D-Glc*p* (1→ glycosyls as the backbone as shown in Figure 6 (15). Furthermore, one acidic-extractable polysaccharide is found to be consisted of Fuc (1.23%), Ribose (0.57%), Ara (0.29%), Xyl (2.12%), Man (2.73%), Gal (4.66%), and Glc (88.4%) (77).

## Bioactivities of *Cordyceps militaris*-Derived Polysaccharides

The polysaccharides obtained from *C. militaris* have been demonstrated to have various biological activities, including antioxidant, immunoregulatory, anti-inflammatory, and anti-tumor activities (7, 12). In the following sections, we discuss the potential structure-activity relationship of these polysaccharides. Furthermore, we summarize the hypolipidemic, anti-diabetic, and anti-atherosclerotic functions of *C. militaris*-derived polysaccharides.

### Anti-oxidation

The antioxidant activity and mechanisms of *C. militaris*-derived polysaccharides have been well documented previously in the literature (3), especially by Gu et al. (7). Here, we discuss the potential structure-activity relationship of these polysaccharides as antioxidants. Xu et al. demonstrated that the monosaccharide composition may significantly influence the antioxidant activity of polysaccharides (78). For instance, polysaccharides from the fruiting body of cultured *C. militaris* grown on silkworm pupae are reported to have better antioxidant activity than that grown on solid rice medium. The former has more Glc and the latter has more Man (79). The purified polysaccharide primarily consisting of Glc (47.5%), Man (34.3%), Gal (10.8%), and Ara (4.85%) in α-type glycosidic linkage can scavenge DPPH, hydroxyl, and superoxide radicals *in vitro* (80). The acidic-extractable heteropolysaccharide mostly composed of Glc (88.4%) not only scavenge free radicals *in vitro* but also improve the activity of antioxidant enzyme in type 2 diabetes mice (77). Furthermore, the mycelia polysaccharide obtained using a weak alkaline (pH 8–9) culture medium is reported to be rich in β-(1→6)-glucan, which has better antioxidant activity than that obtained from weak acidic conditions (pH 5–7) (55). These studies demonstrate that Glc and the β-configuration may enhance the antioxidant activity. Secondly, the ions contained in the polysaccharides may contribute to the antioxidant activity. The polysaccharide-iron (III) composed of →2)-β-D-Glc*p* (1→ and highly branched →2,4)-α-D-Glc*p* (1→ glycosyls shows antioxidant activity almost equal to that of vitamin C. This molecule can scavenge DPPH, hydroxyl, and superoxide anion radicals (54). Similarly, addition of sodium selenite can promote production of Se-polysaccharides with a better antioxidant activity than those without Se. Another study demonstrated that the Se-polysaccharide has better antioxidant activity with regard to scavenging free radicals (81). *In vivo*, Se-polysaccharides can further promote the activity of superoxide dismutase (SOD) (82). Thirdly, the acidic groups may also support the antioxidant activity of these polysaccharides. The acidic polysaccharide mainly consisted of →6) Gal*p* (1→, →4) Glc*p* (1→, and →4,6) Glc*p* (1→ glycosyls and GlcA and GalA can improve the activities of glutathione peroxidase and SOD, thereby reducing malondialdehyde concentration (83). Furthermore, sulfation may enhance the free radical-scavenging effect of polysaccharides as revealed *in vitro* (35). A recent study also indicated that polysaccharides with →2)-α-D-Man*p* (1→ as the backbone exhibit good antioxidant activity (19). Therefore, glucosyls in β-configuration, →2)-α-D-Man*p* (1→ linked backbone, metal ions, and acid groups, may contribute to the antioxidant activity of these polysaccharides.

### Immune Enhancement

**FIGURE 8:**
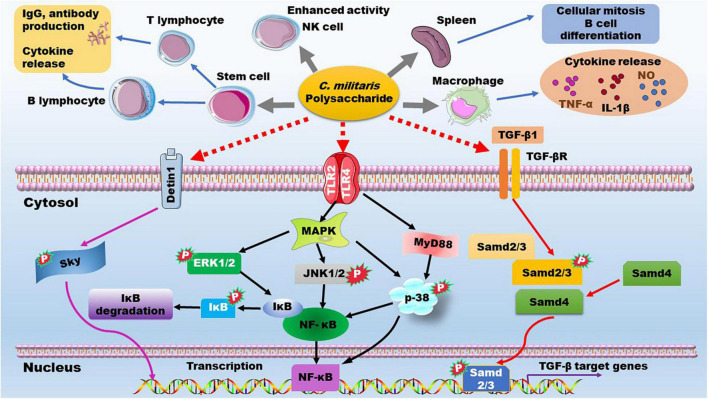
The immune enhancement effects and the underlying mechanisms of *C. militaris*-derived polysaccharides. ERK, extracellular regulated kinase; IgG, immunoglobulin G; IL, interleukin; JNK, C-Jun Kinase; MAPK, mitogen-activated protein kinase; NF-κB, nuclear factor kappa-B; NK cell, natural killer cell; NO, nitric oxide; PRRs, pattern recognition receptors; TGF-β, transforming growth factor β; TGF-βR, TGR-β receptor; TLR, Toll-like receptor; TNF-α, tumor necrosis factor-α.

Immune response plays an importance role in host defense system. B-lymphocyte and T-lymphocyte specifically mediates humoral and cellular immunity, respectively. Polysaccharides from *C. militaris* exhibit mitogenic effects in mouse splenocytes and can promote differentiation of murine T-, B-lymphocytes, and neutrophiles. These polysaccharides also increase IgG function and production of cytokines, such as interleukin (IL)-1β, tumor necrosis factor (TNF)-α, interferon (IFN)-γ, and IL-6 (79, 84, 85). On the cell surface, pattern recognition receptors (PRRs), such as Toll-like receptor (TLR) 2, TLR4, and dectin-1, can trigger different signaling pathways including phosphoinositide-3-kinase/protein kinase B (PKB/AKT), mitogen-activated protein kinase (MAPK), and tyrosine kinases (86, 87). Lipopolysaccharide (LPS) and some polysaccharides can activate these cell surface receptors and exert their immune modulating functions. As recently reviewed by Lee et al. (10), polysaccharides of *C. militaris* are likely to cause type 1 immunity. They can increase TNF-α secretion in macrophages and enhance NK cell activity. The immune enhancement activity of *C. militaris*-derived polysaccharides has been recently reviewed by different groups (3, 7), and Phull et al. have reviewed the influence of monosaccharide composition and Mw on the immunomodulatory effect (12). Here, we consider the related signaling pathways as shown in Figure 8 and discuss the potential structure-activity relationship by focusing on glycosidic linkage.

*C. militaris*-derived polysaccharides that are composed of Glc are reported to act on macrophages and increase production of NO, IL-1β, IL-6 *in vitro* (73). The marker polysaccharide of *C. militaris*, →4)-α-D-Glc*p* (1→ linked glucan, can promote production of NO and cytokines in RAW 264.7 macrophages (70). Mechanistically, this polysaccharide is highly selective for the TLR4/MyD88/p38 axis as demonstrated in TLR4-deficient mice (88). The polysaccharide mainly composed of →4)-α-D-Glc*p* (1→ (∼70%), →4,6)-α-D-Man*p* (1→, and →2,6)-α-D-Gal*p* (1→ glycosyls could promote macrophage phagocytosis and secretion of NO, TNF-α, and IL-6, *via* up-regulation of the MAPK/nuclear factor kappa-B (NF-κB) pathway, including phosphorylation of extracellular regulated kinase (ERK), p-38, and C-Jun Kinase (JNK) (71). The acidic exopolysaccharide mainly composed of →4)-α-D-Glc*p* (1→ can promote cytokine secretion by increasing the phosphorylation of ERK, p-38, and JNK (61). Furthermore, the alkali-extracted polysaccharide CMPB90-1 mainly composed of →6)-α-D-Glc*p* (1→ and →3)-α-D-Glc*p* (1→ glycosyls, is reported to improve macrophage M1 polarization through the activation of TLR2/MAPK/NF-κB signaling pathway (75). These data demonstrate that →4)-α-D-Glc*p* (1→ and →6)-α-D-Glc*p* (1→ glycosyls play key roles in immune enhancement by *C. militaris*-derived polysaccharide *via* the up-regulation of PRRs/MAPK/NF-κB signaling.

Of note, the *C. militaris*-derived glucogalactomannan, whose backbone is composed of →2) Man*p* (1→ and →6) Man*p* (1→ glycosyls, can also interact with PRRs, such as TLR2, TLR4, and dectin-1, and promotes the downstream MAPK/NF-κB signaling pathway, thereby increasing production of NO, reactive oxygen species, and TNF-α, and enhancing phagocytic activity of RAW264.7 macrophages (13, 62, 86). In line with this study, the polysaccharide with →2)-α-D-Man*p* (1→ as its backbone shows immunomodulatory activity by promoting secretion of inflammatory factors by macrophages and inducing macrophage M1 polarization (19). Furthermore, the arabinogalactan-type polysaccharide obtained from the fruiting body of *C. militaris* can promote dendritic cell maturation through activation of TLR4, downstream MAPK signaling (phosphorylation of ERK, p38, and JNK) and NF-κB p50/p65, thereby increasing the expression of IL-12, IL-1β, TNF-α, and IFN-α/β (89). The acidic arabinogalactan-type polysaccharide consists of →5)-Ara*f*-(1→, →4)-Gal*p*-(1→, →4)-GalA*p*-(1→, and Ara*f* (1→ glycosyls can also enhance secretion of inflammatory cytokines and improve production of nitric oxide (NO) by up-regulating the expression of inducible nitric oxide synthase in macrophages (90). Additionally, *C. militaris*-derived β-glucan can enhance macrophage activation and phagocytosis by increasing protein phosphorylation of Lyn, Syk, and MAPK (91).

### Anti-inflammation

The recent review by Phull et al. largely reviewed the immune enhancement activity of *C. militaris*-derived polysaccharides rather than the anti-inflammatory effects (12). Here, we review the anti-inflammatory activity of these polysaccharides based on an analysis of the literature. Some polysaccharides of *C. militaris* are found to suppress secretion of eotaxin, IL-4, IL-5, IL-13, and IFN-γ, and reduce serum IgE level, inflammatory cell infiltration, and goblet cell hyperplasia by inhibiting transforming growth factor β1 (TGF-β1) and the phosphorylation of Smad2/3 proteins in ovalbumin challenged asthmatic mice (92). A recent study demonstrated that cordyceps polysaccharide can reduce acute liver injury by promoting hepatocyte proliferation, liver vascular regeneration, and liver tissue repair in line with the upregulation of vascular endothelial growth factor (VEGF), stromal cell-derived factor-1α, proliferating cell nuclear antigen, and signal regulatory protein α1, and the reduction of IL-18 and caspase-1 (93). Se-rich polysaccharides can effectively reduce inflammation by reducing the mRNA expression of TNF-α and IL-6 as well as serum content of LPS-binding proteins in C57BL/6J mice fed a high-fat diet (94). *C. militaris*-derived β-(1→3)-D-glucan can also inhibit LPS-induced mRNA expression of IL-1β, TNF-α, and cyclooxygenase 2 in THP-1 macrophages and reduce formalin-induced nociceptive response and leukocyte migration (76). Of note, gut microbiota also plays a key role in modulating inflammation. For instance, *Akkermansia* prefers to ingest polysaccharides as its nutritional source, and *A. muciniphila* is reported to suppress intestinal inflammation and improve gut barrier function (95). *C. militaris*-derived polysaccharides can increase the population of *Akkermansia* and *Lachnospiraceae_Eubacterium*, and decrease the abundance of *Bacteroides*, *Parabacteroides*, and *Blautia*, thereby suppressing inflammation (96). A recent study demonstrated that *C. militaris* treatment downregulates the mucosal levels of pro-inflammatory cytokines and upregulates the levels of anti-inflammatory cytokines *via* inhibiting TLR4/MyD88/NF-κB signaling in pigs. Furthermore, this treatment also modulates gut microbiota and increases the concentrations of acetate and butyrate, thereby improving intestinal barrier function (97). Another study demonstrated that Cordyceps improves inflammation *via* modulating the abundance of *Enterococcus cecorum* (98).

### Anti-hyperlipidemia and Anti-atherosclerosis

**FIGURE 9:**
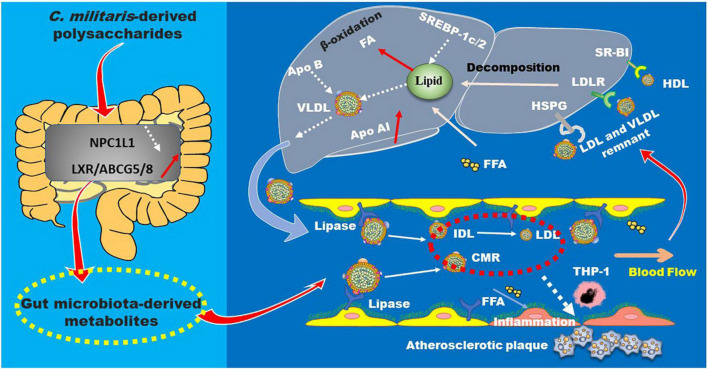
The lipid-lowering mechanisms of *C. militaris*-derived polysaccharides. These polysaccharides are found to modulate multiple genes and proteins related to lipid absorption and metabolism in the plasma, small intestine, and liver, thereby improving hyperlipidemia and atherosclerosis. ABC, ATP-binding cassette; Apo, apolipoprotein; CRM, chylomicron remnant; FFA, free fatty acid; HSPG, heparan sulfate proteoglycan; IDL, intermediate density lipoprotein; LDL, low-density lipoprotein; LDLR, low-density lipoprotein receptor; LRP1, LDLR-related protein 1; LXRα, liver X receptor α; NPC1L1, Niemann-Pick C1-like protein 1; SR-B1, scavenger receptor B type 1; SREBP, sterol regulatory element-binding protein; VLDL, very low-density lipoprotein.

Polysaccharides obtained from mycelia and fruiting body of *C. militaris* can decrease total cholesterol (TC), triglyceride (TG), and low-density lipoprotein (LDL) cholesterol (LDL-C) levels and increase high density lipoprotein cholesterol (HDL-C) in streptozotocin-induced diabetic mice (99, 100). Crude polysaccharides from the fruiting body of *C. militaris* can improve reverse cholesterol transport (RCT) in C57BL/6J mice (101). Furthermore, the →6)-β-D-Glc*p* (1→ linked glucan and the heteropolysaccharide (CM1) primarily consisting of →4)-β-D-Glc*p* (1→, →2)-α-D-Man*p* (1→, and →2,6)-α-D-Man*p* (1→ glycosyls, can improve cholesterol efflux *in vitro* (14). Recently, studies in our group demonstrated that CM1 can alleviate hyperlipidemia and adipocyte differentiation in LDLR*^(±)^* hamsters, whose lipid profiles are similar to human (102). Mechanistically, these polysaccharides are found to up-regulate genes and proteins related to RCT, such as liver X receptors and ATP-binding cassette (ABC) transporters (14, 15, 102). Low-density lipoprotein receptor (LDLR) plays a key role in the clearance of apolipoprotein (apo) B-containing lipoproteins in circulation, and proprotein convertase subtilisin/kexin-type 9 (PCSK9) plays a key role in post-translational degradation of LDLR (103). Our lab obtained a novel alkali-extracted polysaccharide from the fruiting body of *C. militaris* mainly composed of →4)-β-D-Man*p* (1→, →6)-β-D-Man*p* (1→, and →6)-α-D-Man*p* (1→ glycosyls, which can inhibit PCSK9 secretion in Huh7 cells (16). Additionally, Se-rich polysaccharide from *C. militaris* can effectively reduce serum TG and LDL-C by 51.5 and 44.1% in C57BL/6J mice, respectively (94). Mechanistically, this molecule can reduce adiponectin levels and decrease gut bacteria, such as *Dorea*, *Lactobacillus*, *Clostridium*, and *Ruminococcus*, that are negatively associated with obesity. Furthermore, it can increase mucosal beneficial bacteria *Akkermansia*, and has no effect on the content of short-chain fatty acids (94, 104). A recent study demonstrated that Cordyceps may improve obesity *via* modulating the abundance of *Enterococcus cecorum* as well as bile acid metabolism (98). The lipid-lowering mechanisms of these polysaccharides are summarized in Figure 9.

Given that hyperlipidemia promotes the development of atherosclerosis, our group investigated the anti-atherosclerotic effects of the polysaccharides derived from *C. militaris*. As demonstrated in apoE*^(–/–)^* and LDLR*^(–/–)^* mice, the polysaccharide CM1 can significantly decrease atherosclerotic plaque formation in the aorta of these mice. Mechanistically, this molecule can improve the expression of RCT-related genes and proteins in the liver and small intestine of the mice (105). Integrated bioinformatics analysis suggested that these polysaccharides can modulate the expression of hundreds of genes. KEGG and GO enrichment demonstrated that these differentially expressed genes are associated with lipid metabolism, inflammation, oxidation, and shear stress (5). Of importance, these polysaccharides may modulate the lncRNA-miRNA-mRNA axis (6, 85). Additionally, the anti-atherosclerotic effect of these polysaccharides may also be attributed to their effect on the reduction of trimethylamine and oxidized trimethylamine *via* modulating gut bacteria (100). The mannan core and glycosyls in a β-configuration may play an important role in ameliorating atherosclerosis (105, 106). Of note, the TG-lowering effect of *C. militaris*-derived polysaccharides is better than their TC-lowering effect (5, 15, 102, 107). It is known that there is a positive correlation between plasma TG and cardiovascular disease (CVD) (108, 109). These data suggest that *C. militaris*-derived polysaccharides have a potential application in prevention of CVD as TG-lowering compounds.

### Anti-diabetic Activity

In streptozotocin-induced diabetic mice, the mycelia polysaccharide increases body weight and thymus index and decreases fasting blood glucose, insulin resistance as well as secretion of pro-inflammatory cytokines (TNF-α and IL-6) and C-reactive protein (99). The purified polysaccharide CBPS-II with a Mw of 1,273 kDa can reduce blood Glc level in streptozotocin-induced diabetic mice (100). The acid-extracted heteropolysaccharide mainly composed of glucose (88.4%) also showed good hypoglycemic effect in type 2 diabetes mice that were induced by a high-fat diet and streptozotocin (77). Of note, the heteropolysaccharide that is primarily composed of →4)-α-D-Glc*p* (1→, →3,6)-α-D-Man*p* (1→, and →4)-α-D-Man*p* (1→ glycosyls can improve glucose tolerance in STZ-induced diabetic mice, and this activity may be related to an inhibitory effect on alpha-glucosidase (59). Several other labs have also demonstrated that polysaccharides from *C. militaris* can inhibit the activity of alpha-glucosidase (110). It seems that polysaccharides from the fruiting bodies have a better effect on inhibition of alpha-glucosidase than those from fermentation mycelia (65). Furthermore, carboxymethylation and acetylation of these polysaccharides may enhance their inhibitory effects on alpha-glucosidase (111). *C. militaris*-derived polysaccharides can also protect diabetic nephropathy in mice by regulating cell autophagy (112). Additionally, the anti-hyperglycemic effect of *C. militaris*-derived polysaccharides can be partially attributed to its effect on promoting *Akkermansia*, a beneficial bacterium in the gut (96).

### Antitumor Activity

**FIGURE 10:**
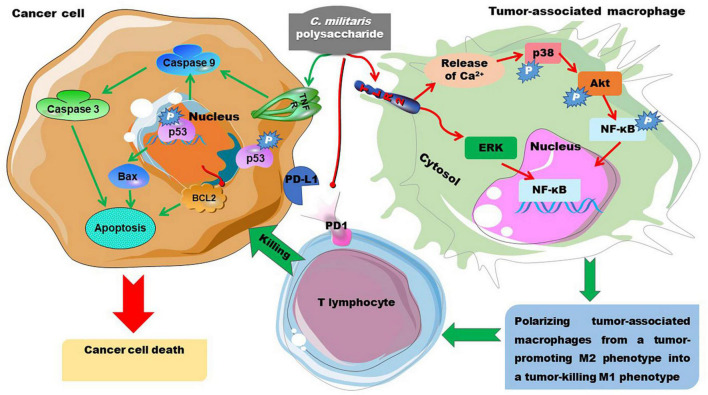
The anti-tumor mechanisms of *C. militaris*-derived polysaccharides. *C. militaris*-derived polysaccharides induce cancer cell death by enhancing apoptosis-associated signaling. Alternatively, they polarize tumor-associated macrophages from M2 to M1 phenotype, thereby promoting the cancer cell killing activity of T lymphocytes. AKT/PKB, phosphoinositide-3-kinase/protein kinase B; Bax, B cell leukemia/lymphoma 2-associated X protein; Bcl-2, B cell leukemia/lymphoma 2; ERK, extracellular regulated kinase; NF-κB, nuclear factor kappa-B; PD-1, programmed death-1; PD-L1, programmed death lignd-1.

The antitumor activity of *C. militaris*-derived polysaccharides has been summarized by Zhang et al. (3) and Gu et al. (7). Here, we have tried to elucidate the potential structure-activity relationship of these polysaccharides. Moreover, the potential antitumor mechanisms of these polysaccharides are summarized in Figure 10. Polysaccharides obtained from mycelia can attenuate doxorubicin-induced cytotoxic effects during chemotherapy (85). Polysaccharides from mycelia (CMPS-II) seem to have a better effect than that obtained from the fruiting body (CBPS-II). CMPS-II and CBPS-II can up-regulate the expression of apoptosis factors including Caspase-3, Caspase-9, and p53, and down-regulating proliferating cell nuclear antigen (64). These two 1,3-branched-galactomannoglucans with triple-helical chains have similar Mw (1.2–1.4 kDa) and the same backbone of →4)-α-D-Glc*p* (1→ glycosyls. However, CMPS-II has significantly more Glc than CBPS-II, suggesting that the branched →4)-α-D-Glc*p* (1→ glycosyls may facilitate the antitumor activity of these polysaccharides. Our team recently demonstrated that the branched glucans primarily consisting of →4)-α-D-Glc*p* (1→ glycosyls can inhibit the proliferation of tumor cells (113). A previous study also demonstrated that water-extracted *C. militaris* polysaccharide, largely composed of →4)-α-D-Glc*p* (1→, →6)-β-D-Glc*p* (1→, and →4)-β-D-Glc*p* (1→ glycosyls, inhibits the proliferation of several tumor cell lines *in vitro* (72). Additionally, a recent study demonstrated that the alkaline-extracted polysaccharides mostly composed of →6)-α-D-Glc*p* (1→ and →3)-α-D-Glc*p* (1→ glycosyls shows anti-tumor effects though the modulation of tumor-associated macrophages, which inhibit the killing effect of T lymphocytes on tumor cells through the programmed death lignd-1/programmed death-1 axis (114). Mechanistically, this molecule binds to TLR2, causes release of Ca^2+^ and activation of p38, AKT, and NF-κB, thereby polarizing tumor-associated macrophages from a tumor-promoting M2 phenotype into a tumor-killing M1 phenotype (114). Furthermore, Se-polysaccharides with a backbone of →4) Glc*p* (1→ glycosyl exhibit an appreciable antitumor effect *in vitro*, and the polysaccharides with a lower Mw (65 and 16 kDa) have better activity than the one with a higher Mw of 1,902 kDa (63). The above data indicate that the glucosyls in the α-D-configuration play a key role in the anti-tumor activity of *C. militaris*-derived polysaccharides.

### Others

Addition of *C. militaris* in diet is reported to improve physical fatigue in mice (115). The crude polysaccharide derived from the fruiting body of *C. militaris* has good anti-fatigue activity (116). One exopolysaccharide exhibits hypouricemic effect in mice by decreasing urate production and the activity of xanthine oxidase (117). The presence of sulfate and polygalacturonic acids endow *C. militaris* polysaccharide with anti-angiogenesis activity *via* reducing the protein expression of VEGF in human umbilical vein endothelial cells (53). A acidic polysaccharide that mainly composed of →6)Gal*p*(1→, →4)Glc*p* (1→, and →4,6)Glc*p*(1→ glycosyls can prevent Pb^2+^-induced liver and kidney toxicity by activating nuclear factor erythroid 2-related factor 2 (Nrf2) signaling pathway and increasing bacterial diversity of gut microbiota (83). Mechanistically, it enhances the protein expression of Nrf2, Kelch-like ECH-associated protein-1, Heme oxygenase, and NAD(P)H: quinone oxidoreductase 1. Furthermore, this polysaccharide promotes the abundance of *Ruminococcaceae* and reduces the abundance of *Lachnospiraceae* families; it reduces *Roseburia* and increases *Bacteroides* genera (83). The exopolysaccharide Cs-HK1 with a high Mw can protect bifidobacterial cells against antibiotics through physical interactions (118). *C. militaris* is found to modulate the formation of short-chain fatty acids (97, 119). Additionally, the acidic arabinogalactan-type polysaccharide obtained from mycelia can reduce virus titer of mice infected with influenza A virus (90). *C. militaris* may also have a potential application for the treatment of mild-to-moderate COVID-19 disease (120).

### Comparisons With Other Studies and What Does the Current Work Add to the Existing Knowledge

Recently, the research involving *C. militaris*-derived polysaccharides has increased rapidly, particularly in their structural characterization and pharmaceutical activities. The pharmaceutical effects of these polysaccharides including antioxidant, immunomodulatory, and anti-tumor activities have been widely reviewed (7, 12). However, the structural characteristics of polysaccharides and the related methods used for elucidating these polysaccharides have not been reviewed in the recent literature. Given that the structure of polysaccharide determines its bioactivity, we describe the methods used for elucidation of polysaccharide structure in this article. Of note, most of the chemical methods are described by presenting the detailed chemical reactions, which are helpful in understanding the principle behind the processes. The development of novel techniques including mass spectrometry are also discussed in this article. Of importance, we review the structural characteristics of *C. militaris*-derived polysaccharides in the format of presumed chemical structure and have discussed the potential structure-activity relationship. The mechanisms of action of these polysaccharides are presented by constructing the signaling pathways as seen in the figures. Furthermore, the anti-diabetic, anti-hyperlipidemic, anti-atherosclerotic, and gut microbiota modulatory effects that have not been elucidated well in previous reviews in the literature are also summarized in this article. This detailed review focuses on polysaccharide structure and bioactivity and makes it possible to discuss and understand the structure-activity relationship of *C. militaris*-derived polysaccharides. It is found that different glycosyls and functional groups may play distinct roles in the bioactive functions of these polysaccharides.

## Conclusion and Future Perspective

The widespread use of advanced analysis tools such as HPLC, MS, and NMR techniques has greatly improved the structural elucidation of *C. militaris*-derived polysaccharides. Most of the reported heteropolysaccharides have mannosyls, such as →2)-α-D-Man*p* (1→, →6)-α-D-Man*p* (1→, or →6)-β-D-Man*p* (1→ glycosyls, as their core. The obtained glucans are found to consist of →4)-α-D-Glc*p* (1→, →6)-α-D-Glc*p* (1→, or →3)-β-D-Glc*p* (1→ glycosyls as their backbone. Recently developed novel techniques, such as mass spectrometry imaging and the novel NMR methods, are definitely going to improve our understanding of the structure of *C. militaris*-derived polysaccharides in the future. *C. militaris*-derived polysaccharides can modulate multiple signaling pathways and have great potential for use as dietary supplements and health food products for the prevention and treatment of oxidation, inflammation, tumors, immune dysfunction, and metabolic syndrome. The glucosyls in a β-configuration, →2)-α-D-Man*p* (1→ linked backbone, metal ions, and acid groups, may all contribute to the antioxidant activity of these polysaccharides. The α-D-glucosyls and α-D-mannosyls mainly contribute to the immune enhancing activity, while the β-D linked glycosyls and α-D-mannosyls may facilitate the hypolipidemic and anti-atherosclerotic effects. Additionally, the branched →4)-α-D-Glc*p* (1→, →6)-α-D-Glc*p* (1→, and →3)-α-D-Glc*p* (1→ glycosyls may enable the anti-tumor effect of *C. militaris*-derived polysaccharides.

The high-frequency degeneration of *C. militaris* during cultivation has limited the development of the *C. militaris* industry (9). Gene engineering strategies are expected to further improve the yield of *C. militaris*-derived polysaccharides. The structural characteristics, including Mw, monosaccharide composition, glycosyl linkage, glycosyl configuration, physicochemical properties, and even the animal models and cultivation conditions of *C. militaris* may influence their bioactivity. Therefore, standardized procedures are needed to guarantee the quality of *C. miliatris*-derived polysaccharides. The focus of the standardization procedures should be on cover strain preservation, cultivation conditions, extraction and purification methods, quality control, and impurity detection. The recent studies using purified polysaccharides have greatly improved our understanding of their structure-activity relationships. However, we still do not know the functional groups and/or bioactive domains due to lack of comparative studies. To improve this, researchers may design parallel experiments to evaluate the activity and/or the underlying mechanisms of polysaccharides of interest containing different backbones or differently digested polysaccharides with the same backbone or polysaccharides with different chemical modifications. Furthermore, given the large Mw of these polysaccharides, they may have little change of directly entering the circulation. One possible mechanism is that these molecules can exert their bioactivity *via* modulating gut microbiota and their metabolites, which can easily penetrate the intestinal barrier and then work within the circulation. Another possibility is that the polysaccharides digested by the gastric acid or gut microbiota with a lower Mw or the corresponding oligosaccharides may be directly absorbed by the intestine, thereby exerting their bioactivities in different organs. The polysaccharides that are resistant to degradation *in vivo* may either act directly on receptors in the gut or work through mechanisms involving physical binding. These ideas need to be investigated further in future studies.

## Author Contributions

MM, W-QY, YL, and Y-LS performed reference collection. MM, W-QY, and YL drew the figures. S-DG wrote and re-edited the manuscript. All authors contributed to the article and approved the submitted version.

## Conflict of Interest

The authors declare that the research was conducted in the absence of any commercial or financial relationships that could be construed as a potential conflict of interest.

## Publisher’s Note

All claims expressed in this article are solely those of the authors and do not necessarily represent those of their affiliated organizations, or those of the publisher, the editors and the reviewers. Any product that may be evaluated in this article, or claim that may be made by its manufacturer, is not guaranteed or endorsed by the publisher.

## Footnotes

1
https://glygen.ccrc.uga.edu/ccrc/specdb/ms/pmaa/pframe.html

## Abbreviations

ABC: ATP-binding cassette
apo: apolipoprotein
Ara: arabinose
CM1: heteropolysaccharide
COSY: correlated spectroscopy
CVD: cardiovascular disease
DEPT: polarization transfer spectroscopy
ERK: extracellular regulated kinase
ESI-MS: electrospray ionization mass spectrometry
FT-IR: Fourier transform infrared
Gal: galactose
GalA: galacturonic acid
GC: gas chromatography
GC-MS: gas chromatography-mass spectrometry
Glc: glucose
GlcA: glucuronic acid
HDL-C: high density lipoprotein cholesterol
HILIC-MS: Hydrophilic interaction chromatography coupled to mass spectrometric detection
HMBC: heteronuclear multiple bond correlation spectroscopy
HMQC: heteronuclear multiple quantum coherence spectroscopy
HPLC: high-performance liquid chromatography
HPLC-MS/MS: HPLC-tandem mass spectrometry
IFN: interferon
IL: interleukin
JNK: C-Jun Kinase
LDL: low-density lipoprotein
LDL-C: LDL cholesterol
LDLR: Low-density lipoprotein receptor
LPS: Lipopolysaccharide
MALDI: matrix-assisted laser desorption/ionization
MALDI-TOF: MALDI-time of flight
Man: mannose
MAPK: mitogen-activated protein kinase
MS: mass spectrometry
Mw: molecular weight
NF-κB: nuclear factor kappa-B
NMR: nuclear magnetic resonance
NO: nitric oxide
NOESY: nuclear overhauser effect spectroscopy
Nrf2: nuclear factor erythroid 2-related factor 2
PCSK9: proprotein convertase subtilisin/kexin-type 9
PKB/AKT: phosphoinositide-3-kinase/protein kinase B
PMP: 1-phenyl-3-methyl-5-pyrazolone
PRR: pattern recognition receptor
RCT: reverse cholesterol transport
SOD: superoxide dismutase
TC: total cholesterol
TFA: trifluoroacetic acid
TG: triglyceride
TGF-β1: transforming growth factor β1
TLR: Toll-like receptor
TNF: tumor necrosis factor
TOCSY: total correlation spectroscopy
VEGF: vascular endothelial growth factor.
